# Network analysis of the relationship between stress coping mechanisms and performance in high school women's volleyball competitions

**DOI:** 10.3389/fspor.2026.1701323

**Published:** 2026-01-30

**Authors:** Feng Li, Dewei Mao

**Affiliations:** 1 Qufu Normal University, Qufu, China; 2 Shandong Sport University, Jinan, China

**Keywords:** competition performance, coping strategies, network analysis, psychobiosocial experiences, recovery-stress balance, sports emotions

## Abstract

This study aims to explore the coping mechanisms of high school female volleyball players, including the associations between four factors (strategies, psychobiosocial experiences, sports emotions, and recovery-stress balance) and competition performance. A total of 96 valid participants were recruited from the girls’ division of the 1st National Secondary School Volleyball Regional League Finals, and their psychological states were assessed using the Athletic Coping Skills Inventory (ACSI-28), Psychobiosocial Experience Semantic Differential Scale in Sport (PESD-Sport), Sport Emotion Questionnaire (SEQ), and Recovery-Stress Questionnaire for Athletes (RESTQ-Sport). Network analysis was adopted to analyze the relationships between the four factors and competition performance: (1) Confidence and achievement motivation (EI = 1.207, bridge EI = 0.423) is identified as the most influential nodes in the network and simultaneously function as bridge nodes, linking multiple communities. In addition, dejection (weight = 0.146), sports recovery (weight = −0.097), (2) and goal setting/mental preparation (weight = 0.048) are closely associated with competition performance, with dejection showing the strongest connection to competition performance. These findings yield empirical, data-driven implications for coaches and sport psychologists, indicating that targeted interventions designed to boost athletes’ confidence and achievement motivation may generate comprehensive benefits spanning emotional regulation and competitive performance. Moreover, the proactive monitoring and regulation of negative affective states (e.g., dejection) could facilitate more precise psychological interventions, thereby optimizing the competitive performance of female high school volleyball players.

## 1 Introduction

As sports evolve and the level of competition increases, training tailored to the psychological state of volleyball players can enhance their performance and lead to positive outcomes. High-intensity training, frequent matches, risk of injury, and performance expectations can negatively impact the physiology and psychology of high school volleyball players. Without effective stress management, these pressures may reduce athletic performance or even cause psychological issues (1). Therefore, athletes’ psychological coping skills, particularly cognitive and behavioural strategies to manage training and competition stress, are crucial for enhancing competitive performance and maintaining psychological well-being (2, 3). Despite the growing body of research on stress coping skills and psychological adjustments in athletes, effectively integrating these psychological factors and exploring their relationship with competition performance remains a complex and urgent issue (2).

Athletes’ competitive performance is shaped by the interplay of physiological, psychological, and socio-environmental factors. The Individual Zones of Optimal Functioning (IZOF) model highlights that these factors intersect dynamically when facing athletic challenges (4), while Morano et al. (5) noted that self-esteem and athleticism indirectly affect burnout through functional psychobiosocial experiences.Existing research has focused on the relationship between competition performance and multiple stress coping mechanisms; however, the dynamics among various stress coping factors and competition, as well as multiple psychological factors, remain unclear. Network analysis enables simultaneous consideration of multiple constructs, exploring their interrelationships and the potential for stable structures. By constructing a network, we identify the most influential constructs (6). This study incorporates multiple factors related to stress coping, psychobiosocial experiences, and competition performance, using network analysis to examine the constructs associated with competition outcomes and determine which are more strongly associated with performance and exert a greater influence on the overall psychological state of athletes (7).

### 1.1 Stress coping-recovery and competition results

Psychological stress in athletes can arise from pre-competition expectations, in-event challenges, or post-competition frustrations, and effective stress management is intricately linked to competition performance (8). Sport coping skills involve cognitive and behavioral strategies that athletes employ to manage stress, challenges, and frustrations in training and competition (9–11). Recent research has increasingly focused on motor coping skills. For example Christensen and Smith (12), investigated the predictive role of psychological coping skills in collegiate golfers’ game performance and demonstrated that skills such as goal setting, emotion regulation, and confidence were significantly associated with better outcomes. Additionally, coping skills are crucial in athletes’ career development, aiding them in adjusting their mindset and behavior to overcome professional hurdles (13). Nicholls et al. (14) analyzed English rugby union players’ coping strategies and discovered a significant correlation with mental toughness, optimism, and pessimism: the use of positive strategies, such as goal setting and emotion regulation, enhanced performance under pressure.

Additional studies have examined the application of coping skills across various sports. Kristiansen et al. (15) assessed coping strategies in top Norwegian athletes, finding that psychological coping skills like focus adjustment and emotional management had diverse effects depending on the sport but generally benefited athletes’ mental health and competitive performance. Fogaca (16) reported that a combined intervention of coping strategies and social support substantially boosted student-athletes’ mental health and competition performance. Effective Coping strategies can help athletes better manage the stress of competition and training. Nieuwenhuys et al. (17) observed that elite athletes employing effective coping strategies reported higher success rates in well-performed competitions, indicating a greater alignment between their coping strategies and their meta-experience, suggesting that congruence with self-reflections and emotional appraisals leads to enhanced performance.

Besides coping skills, the concept of recovery-stress is pivotal in maintaining athletes’ physical and mental health and excelling in performance. Recovery-stress describes how individuals restore or maintain health through recovery processes when confronted with stress (18). Laux et al.’s (18) study found a significant relationship between stress, recovery, and the increased risk of injury in football players, with notable differences in recovery perception between genders and sport types (team vs. individual) (19). Barnett (20) emphasized the importance of balancing training, competition stress, and recovery. Kellmann and Beckmann (21) argued that comprehensive recovery is essential for maintaining functional fitness and sustained performance in elite sports.

Therefore, to thoroughly examine the relationship between volleyball players’ stress coping, recovery, and game performance, we utilized stress coping skills and recovery balance as operational indicators. This approach allowed us to explore the psychological dimensions of volleyball players’ stress related to the game and identify critical intervention indicators for stress coping.

### 1.2 Psychobiosocial experiences and competition performance

Psychobiosocial experiences, originally conceptualized in medicine to explain the multidimensional causes of disease (22), are now employed in sport psychology to understand athletes’ performance and recovery processes. The Individual Optimal Functional Area (IOFA) model, a sport-specific framework developed by psychologist Hanin in the 1970s, illustrates the relationship between emotional experience and relative success in sport tasks on an individual basis. This model highlights that an athlete's performance is not the result of a single factor but arises from complex interactions among physiological, psychological, and social environments (4). Within the IZOF model framework, psychobiosocial experience is viewed as a subjective manifestation of both emotion and non-emotion in performance-related (state-based) or relatively stable (trait-based) scenarios, reflecting overall human functioning (23).

Psychobiosocial experiences encompass interrelated patterns including enjoyment, confidence, anxiety, motivation, will, assertiveness, and cognitive components (psychological), alongside bodily-somatic and motor behavior (biological), operational, communication, and cognitive aspects (4, 24–26). Empirical evidence confirms their relevance: self-esteem and motor competence indirectly influence burnout via functional psychobiosocial experiences [with gender differences observed; (5)], while harmonious and obsessive passion exert positive indirect effects through group management skills (27). For taekwondo athletes, self-confidence correlates positively with functional psychobiosocial experiences, worry correlates negatively, and pleasurable emotions align with most dimensions of these experiences (28).

Emotions play a critical role in psychobiosocial experiences. Although measures related to sport include enjoyment, confidence, and anxiety, they fall short of capturing the broader spectrum of typical sports-related emotions. Sports emotions, defined as the emotional state athletes experience before a competition (29, 30), have been linked to optimal and suboptimal performance states. Robazza et al.'s (31) research found that athletes perform best in their unique optimal emotional and physical states, and poorly in adverse states. Furthermore, studies have shown that anger can enhance muscle peak force performance in football players (32), and impairment in emotional self-regulation resources can detrimentally affect competition performance (33). Robazza et al. (34) study indicated that cognitive mastery through cognitive reappraisal positively affects sports emotions and psychobiosocial experiences indirectly. Autonomy and belongingness need satisfaction also had positive indirect effects on pleasant emotions (happiness and excitement) and most dimensions of psychobiosocial experiences through cognitive reappraisal (35). In summary, emotions have a non-negligible role between Psychobiosocial experiences and Competition performance in athletes.

Therefore, to more comprehensively explore the role of psychobiosocial experiences in athletes' competition performance, we have included measures of sports psychobiosocial experiences and refined measurements of sports emotions to elucidate their relationship with competition outcomes.

Due to the unique nature of sport competitions, it is essential to delineate the type of sport, age, and gender of athletes more finely to obtain precise and specific intervention training recommendations (36). Volleyball, as a collaborative sport, provides insight into the combined effects of individual psychological states and teamwork on competition performance in high-pressure situations. Moreover, the broad applicability and multidisciplinary intersectionality of volleyball research hold significant practical relevance for both professional athletes and amateur groups. Given that high school girls’ volleyball players are in a phase of rapid physiological and psychological development (37), in-depth research into their psychological states related to volleyball competition is crucial for developing targeted training programs and facilitating career development.

In summary, this study aims to construct a network system that influences the competition performance of Chinese high school female volleyball players through network analysis. The network “node” includes dimensions of Recovery Stress (RESTQ), Athletic Coping Skills (ASCI), Psychobiosocial Experiences (PESD), Sports emotions (SEQ), and Competition performance to explore the factors affecting their competition outcomes. The connectivity of nodes in a network reflects the links between observed variables (38). Based on existing research, this study intends to investigate:

(1) How are the dimensions of RESTQ, ACSI, PESD, and SEQ interconnected, and what are their relationships with competition performance?
(2) In the constructed network, what are the edge weights between nodes, which nodes serve as core elements, and what is the accuracy and stability of the network?

## 2 Research methodology

### 2.1 Research objectives

Convenience sampling was employed in this study. The survey participants were athletes from the girls’ division of the finals of the 1st National Volleyball Regional League for Secondary School Students. The research team distributed questionnaires uniformly during the competition, clearly informing the athletes and team coaches about the purpose of the study, the use of the data, and the confidentiality principles. All participants voluntarily completed the questionnaires. Out of 105 participants who completed all questionnaires, 96 valid questionnaires were obtained after excluding those with omitted or overly regular answers, resulting in an effective recovery rate of 91.42%. The average age of the respondents was 17.26, and they were all informed that their responses would be used for scientific research, adhering to the principle of confidentiality.

### 2.2 Research tools

#### 2.2.1 Athletic coping skills

The Athletic Coping Skills Inventory (ACSI-28), compiled by Miranda et al. (39), was used to assess the thoughts and actions athletes employ to manage internal or external sport-related demands (65). This inventory consists of 28 items on a 4-point scale (0 = “almost never”, 3 = “almost always”) and encompasses seven dimensions: Coping with Adversity, Peaking Under Pressure, Goal Setting/Mental Preparation, Concentration, Freedom from Worry, Confidence and Achievement Motivation, Coachability. The Cronbach's alpha coefficient for this study was 0.897.

#### 2.2.2 Psychobiosocial experiences

The Psychobiosocial Experience Semantic Differential scale in sports (PESD-Sport), developed by Robazza et al. (40), includes 30 items to assess psychological, physical, and social patterns. The psychological dimension contains five subscales: Emotion, Confidence, Anxiety, Assertiveness, and Cognitive. The biological dimension includes Physical and Motor Behavior items. The social dimension consists of three subscales: Operational, Communicative, and Social Support. Each item is represented by an adjective and its antonym on a 9-point bi-directional Likert scale, ranging from −4 (very much in line) to 0 (neutral) to indicate “dysfunction,” and from 0 to 4 to indicate “normal functioning”. The Cronbach's *α* coefficient in this study was 0.978.

#### 2.2.3 Sports emotions

The Sport Emotion Qusetionare (SEQ), developed by Jones et al. (29), measures five dimensions of athlete emotions prior to competition—Anxiety, Dejection, Anger, Excitement, and Happiness—across 22 items on a 5-point scale (0 = not at all, 4 = completely). The Cronbach's alpha coefficient for this study was 0.826.

#### 2.2.4 Recovery-stress

The Recovery-Stress Questionnaire for Athletes, developed by Kellmann and Kallus (41), was used. The questionnaire consists of 76 items, each rated on a 7-point scale (0 = “never”, 6 = “always”). These 76 items are divided into 19 scales, which are grouped into four dimensions (Kellmann & Kallus, 2016): seven scales assess General Stress (GS), five scales assess General Recovery (GR), three scales measure Sport Stress (SS), and four scales measure Sport Recovery (SR). General Recovery and Sport Recovery together constitute the Total Recovery factor, while GS and Sport Stress together form the Total Stress factor. The Recovery-Stress Balance (RSB) dimension is calculated by subtracting Total Stress from Total Recovery. A positive RSB value indicates a balanced relationship between recovery and stress. The Cronbach's *α* coefficient in this study was 0.909.

#### 2.2.5 Competition performance

The competition results of each sports team were used as an indicator of competition performance. Athlete competition result scores were derived using the common scoring method employed by the Chinese Volleyball Association (Appendix A).

### 2.3 Data analysis

Descriptive statistics and Pearson correlation analyses were performed using SPSS 26. Network analysis was conducted following the reporting criteria for psychological network analysis in cross-sectional data (42). The network was estimated using the R package bootnet (43). Networks were visualized in R (version 4.1.4) using the plot function in the bootnet package, which relies on a partial correlation matrix to estimate the relationships between variables included in the model. To reduce spurious correlations, the Graphical Least Absolute Shrinkage and Selection Operator (GLASSO) was used to obtain a regularized network for visualization. In this study, positively correlated edges are shown in blue and negatively correlated edges in red. Thicker edges indicate stronger connections between nodes, with edge weights representing the specific value of the correlation. In networks, nodes that are clustered together have stronger connections with each other (7). To achieve the cleanest model, the recommended tuning parameter of 0.25 was used (43).

Expected Influence is calculated and visualised using the centralityTable and centralityPlot functions in the qgraph package (44). Expected Influence is the sum of the weights of edges connected to a node. A higher Expected Influence index indicates a more influential node in the network, with EI representing the specific value of Expected Influence. Unlike traditional centrality measures, edge weights less than 0 are not taken to their absolute value when calculating Expected Influence; rather, their sign is preserved to distinguish between positive and negative relationships, offering a more comprehensive assessment of influence (45). Although other centrality measures, such as intensity, closeness, and betweenness, exist, only Expected Influence was examined in this study, as recent literature suggests that these other measures may be less applicable to psychological networks than to social networks (46, 47).

Predictability for each node is estimated using the R package mgm (48). Predictability refers to “the degree to which a given node can be predicted by all other nodes in the network” (49). It is graphically displayed as a circle around the node and is an absolute measure of interconnectivity, reflecting the variance of a node explained by its neighbours. Other measures of network structure, such as Strength Centrality (50) and Expected Influence (45), are often used in network literature, but these focus only on the relative importance of nodes. Therefore, the node interconnection problem is addressed using nodePredictable (51).

Two bootstrapping methods, implemented in the R package bootnet, were used to assess the accuracy and stability of the network parameters (7). Firstly, 95% confidence intervals (CIs) for the edge weights were computed through non-parametric bootstrapping with 1,000 iterations to determine accuracy. Second, correlation stability (CS) coefficients were estimated using corStability to assess the stability of centrality index ordering. Simulation studies have shown that CS coefficients greater than 0.25 indicate moderate stability, and coefficients greater than 0.50 indicate strong stability (7). Edge weight difference tests and centrality difference tests were performed to assess whether edge weights or node centrality differ significantly (7).

## 3 Findings

### 3.1 Common methodological biases

SPSS 26.0 software was used for statistical data processing. The common method bias was assessed using the Harman one-way method, which extracted 18 common factors with eigenvalues greater than 1. The first common factor explained 35.02% of the total variance, which is below the 40% threshold, indicating no significant common method bias.

### 3.2 Descriptive statistics and correlation analysis

The means, standard deviations, and correlations for each variable dimension are shown in Table 1.

**Table 1:** Results of descriptive statistics and correlation analysis for each variable.

h	M	SE	1	2	3	4	5	6	7	8	9	10	11	12	13	14	15	16	17	18	19	20	21	22	23	24	25	26	27	28
Grade	7.521	3.016																												
ACSI_1	1.516	0.474	−0.087																											
ACSI_2	1.201	0.402	0.121	0.118																										
ACSI_3	1.698	0.588	−0.019	.678**	0.089																									
ACSI_4	1.807	0.589	−.201*	.676**	0.187	.769**																								
ACSI_5	1.529	0.626	0.053	.661**	.225*	.704**	.718**																							
ACSI_6	1.365	0.545	−0.032	.566**	0.194	.669**	.625**	.711**																						
ACSI_7	1.443	0.341	0.086	0.042	−0.107	−.212*	−0.187	−0.149	−.212*																					
PESD_1	2.028	1.740	−.262*	.323**	−0.014	.401**	.504**	.406**	.366**	−.373**																				
PESD_2	1.938	1.828	−.280**	.354**	0.014	.419**	.535**	.419**	.409**	−.324**	.902**																			
PESD_3	1.118	1.755	−0.085	.262**	0.062	.338**	.348**	.315**	.443**	−0.164	.670**	.741**																		
PESD_4	2.122	1.756	−.313**	.394**	−0.022	.508**	.522**	.420**	.350**	−.285**	.796**	.883**	.637**																	
PESD_5	1.969	1.722	−.289**	.397**	−0.073	.516**	.493**	.425**	.327**	−.279**	.772**	.835**	.638**	.958**																
PESD_6	1.635	1.842	−0.148	.394**	−0.114	.507**	.531**	.412**	.350**	−.335**	.819**	.802**	.693**	.762**	.779**															
PESD_7	1.757	1.745	−.202*	.421**	−0.091	.557**	.532**	.470**	.407**	−.240*	.783**	.827**	.730**	.871**	.893**	.828**														
PESD_8	1.538	1.722	−0.187	.362**	−0.084	.515**	.508**	.459**	.467**	−.229*	.774**	.864**	.786**	.802**	.802**	.770**	.892**													
PESD_9	1.955	1.744	−.287**	.351**	0.042	.455**	.530**	.380**	.350**	−.330**	.857**	.868**	.726**	.849**	.840**	.784**	.845**	.790**												
PESD_10	1.802	1.849	−.208*	.305**	−0.026	.461**	.537**	.419**	.373**	−.341**	.884**	.907**	.688**	.814**	.805**	.784**	.803**	.815**	.885**											
SEQ_1	1.023	0.773	.291**	−.209*	−0.130	−.331**	−.308**	−0.133	−.340**	.297**	−.282**	−.288**	−.243*	−.296**	−.311**	−.304**	−.295**	−.286**	−.343**	−.311**										
SEQ_2	0.721	0.776	.458**	−0.163	0.014	−.225*	−.266**	−0.090	−.257*	.323**	−.313**	−.275**	−0.190	−.245*	−.251*	−.315**	−.255*	−.211*	−.301**	−.287**	.793**									
SEQ_3	2.578	1.007	−0.163	.290**	0.023	.402**	.484**	.309**	.328**	−0.167	.285**	.268**	0.170	.307**	.254*	.407**	.288**	.243*	.219*	.206*	−.315**	−.349**								
SEQ_4	0.792	0.880	.364**	−.202*	0.118	−0.180	−0.120	0.006	−0.143	.269**	−.291**	−0.197	−0.068	−0.139	−0.165	−.279**	−0.181	−0.134	−.272**	−.232*	.615**	.807**	−0.153							
SEQ_5	2.563	1.018	−0.185	.310**	0.011	.433**	.534**	.358**	.378**	−0.154	.319**	.306**	0.159	.346**	.289**	.401**	.329**	.259*	.263**	.257*	−.405**	−.386**	.921**	−0.186						
RESTQ_1	2.190	0.971	0.075	−.282**	.271**	−.407**	−.359**	−.205*	−.320**	.351**	−.491**	−.443**	−.336**	−.392**	−.387**	−.657**	−.431**	−.427**	−.408**	−.431**	.368**	.321**	−.406**	.409**	−.400**					
RESTQ_2	2.115	1.164	0.114	−.264**	.315**	−.368**	−.332**	−0.176	−0.162	.241*	−.442**	−.395**	−.236*	−.407**	−.406**	−.644**	−.450**	−.403**	−.364**	−.368**	.289**	.271**	−.368**	.315**	−.364**	.871**				
RESTQ_3	3.398	0.868	−.235*	.496**	0.067	.610**	.731**	.549**	.459**	−.234*	.612**	.561**	.355**	.564**	.574**	.637**	.584**	.507**	.599**	.602**	−.370**	−.347**	.489**	−.224*	.562**	−.369**	−.392**			
RESTQ_4	7.353	1.027	−.277**	.524**	0.022	.656**	.704**	.553**	.435**	−0.183	.563**	.586**	.334**	.613**	.619**	.620**	.622**	.530**	.583**	.579**	−.348**	−.297**	.470**	−0.197	.510**	−.339**	−.414**	.849**		
RESTQ_5	3.222	1.633	−.212*	.475**	−0.168	.620**	.641**	.443**	.411**	−.310**	.643**	.606**	.384**	.604**	.607**	.789**	.639**	.572**	.593**	.601**	−.420**	−.378**	.530**	−.356**	.559**	−.812**	−.849**	.782**	.788**	

* *p* < 0.05.

** *p* < 0.01.

### 3.3 Network analysis

#### 3.3.1 Network estimation

**Figure 1:**
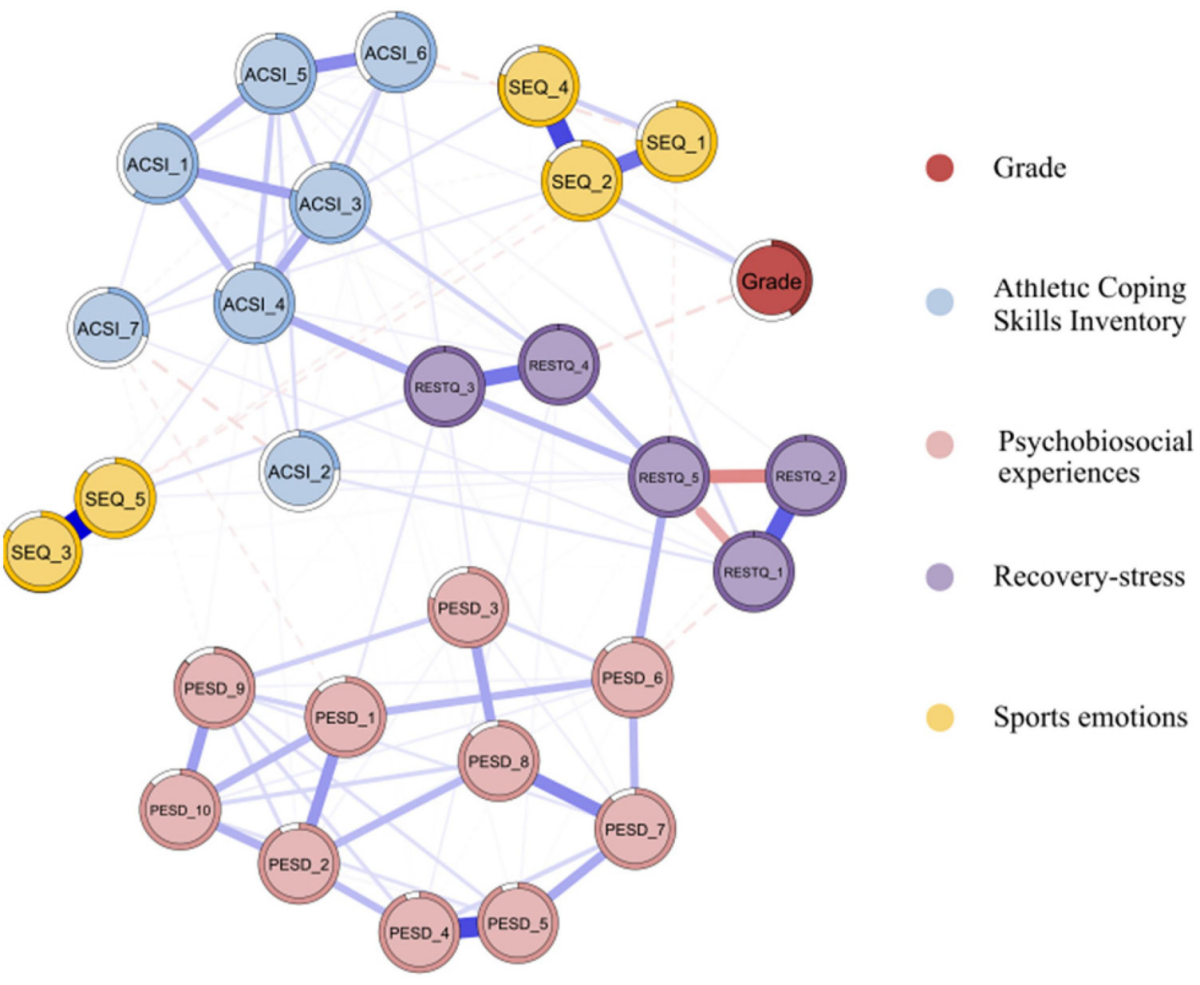
Network structure of competition result, athletic coping skills, psychobiosocial experiences, recovery-stress, and sports emotions. Blue edges indicate positive bias correlation, red edges indicate negative bias correlation, and thick lines indicate stronger connections. Grade = competition result, ACSI_1 = ACSI_Coping With Adversity, ACSI_2 = ACSI_Coachability, ACSI_3 = ACSI_concentration, ACSI_4 = ACSI_Confidence and Achievement Motivation, ACSI_5 = ACSI_Goal Setting/Mental Preparation, ACSI_6 = ACSI_Peaking Under Pressure, ACSI_7 = ACSI_Freedom From Worry, PESD_1 = PESD_Emotion, PESD_2 = PESD_Confidence, PESD_3 = PESD_Anxiety, PESD_4 = PESD_Assertiveness, PESD_5= PESD_Cognitive, PESD_6 = PESD_Bodily-somatic, PESD_7 = PESD_Motor-behavioural, PESD_8 = PESD_Operational, PESD_9 = PESD_Communicative, PESD_10 = PESD_Social support, SEQ_1 = SEQ_Anxiety, SEQ_2 = SEQ_Dejection, SEQ_3 = SEQ_Excitement, SEQ_4 = SEQ_Anger, SEQ_5 = SEQ_Happiness, RESTQ_1 = General stress, RESTQ_2 = Sport Stress, RESTQ_3 = General Recovery, RESTQ_4 = Sport Recovery, RESTQ_5 = Recovery-stress balance. Node circle describes the Predictable for that particular node.

A Gaussian graph-theoretic model was applied to estimate the network structure of Competition Results, RESTQ, ASCI, PESD, and SEQ (Figure 1). The network consisted of 121 significant edges (non-zero weights), which exhibited moderate sparsity relative to the possible 378 edges. The average weight of these edges was 0.276. The average Predictability for all nodes in the network was 0.813, indicating that 81.3% of the variance in a node can be explained by other nodes in the network. A description of each node is provided in Figure 1. The network contains 28 nodes, with different dimensions of the same variable clustering together more frequently, suggesting that nodes within a variable are more closely connected.

#### 3.3.2 Centrality indicators

**Figure 2:**
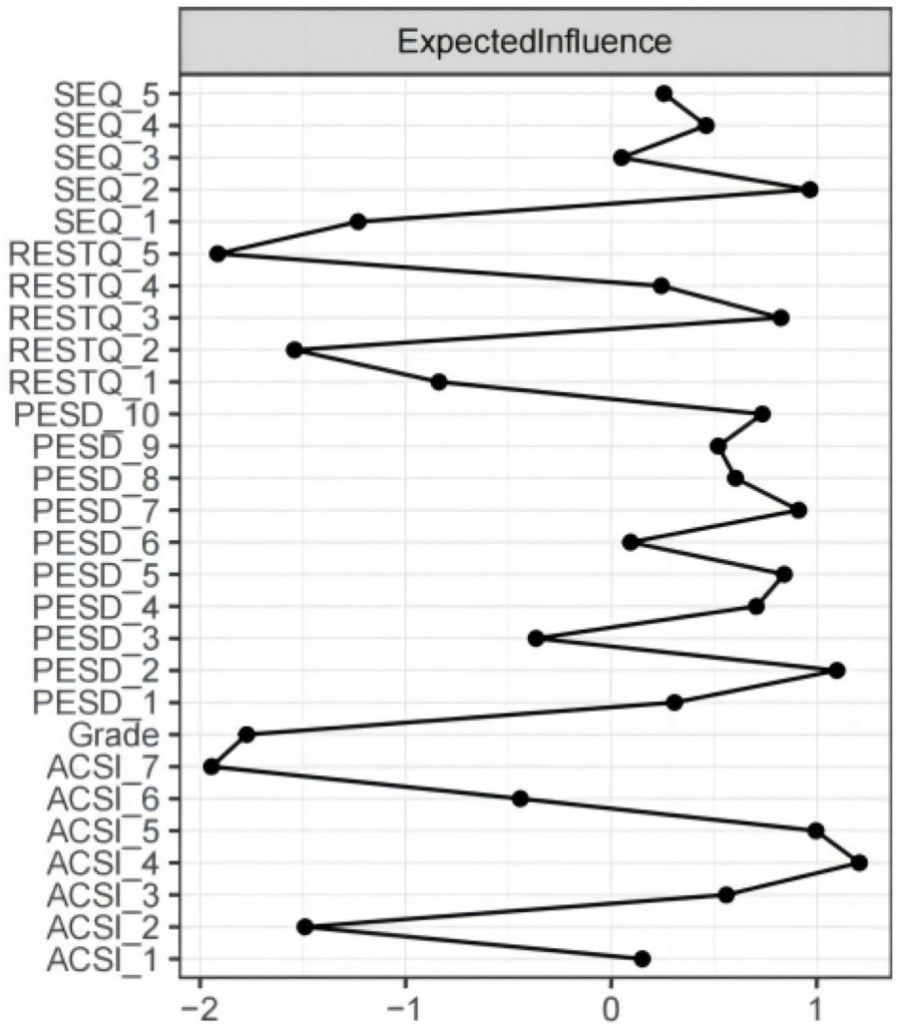
Expected influence index.

**Figure 3:**
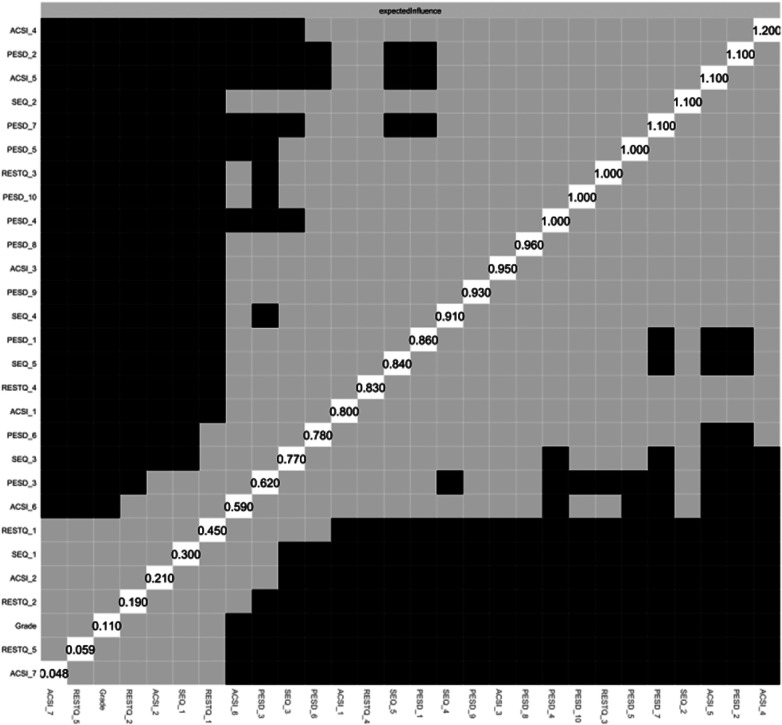
Test of difference for expected influence.

The Expected Influence index was used as a centrality measure for the network nodes (Figure 2). In this network, ASCIConfidence and Achievement Motivation (EI = 1.207) had the highest Expected Influence, followed by PESDConfidence (EI = 1.098), ACSIGoal Setting/ Mental Preparation (EI = 0.999), and SEQDejection (EI = 0.967). These nodes exert a greater influence on the network. A comparison of the Expected Influence variance is shown in Figure 3, where the grey box represents a statistically non-significant difference in the edge weights of the two corresponding edges, and the black box indicates a statistically significant difference.

**Figure 4:**
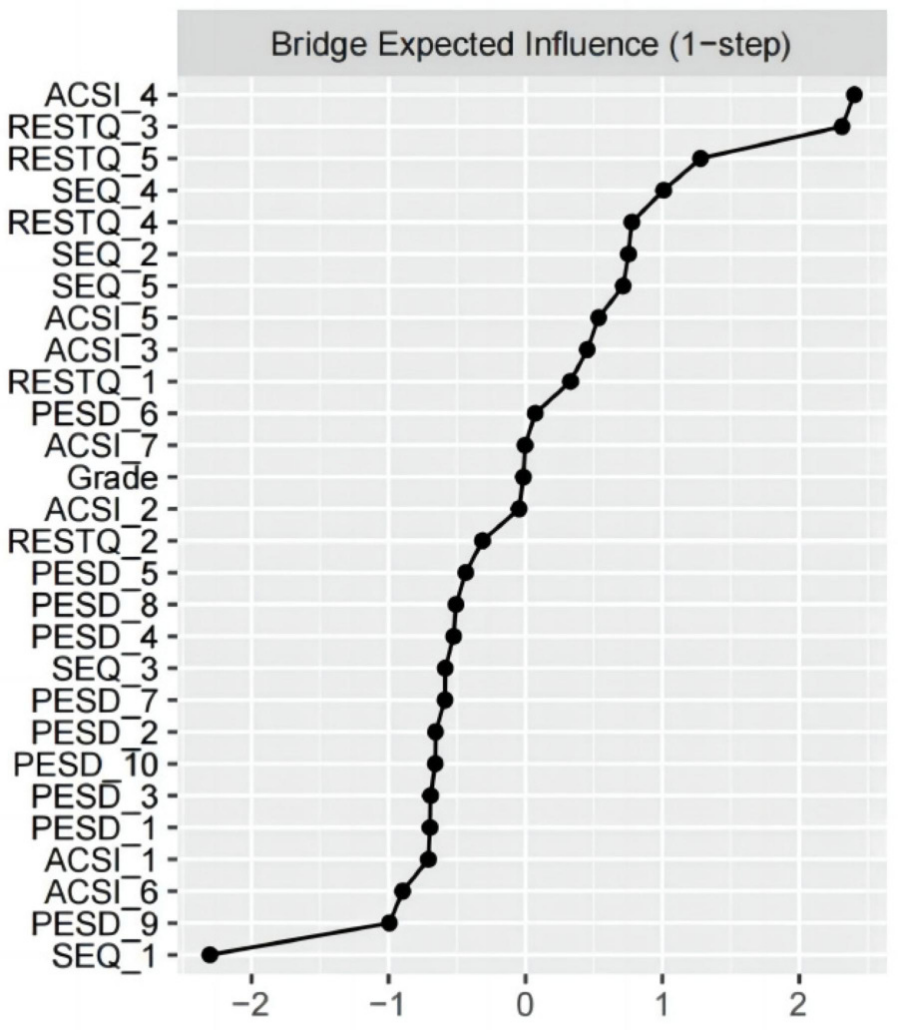
Bridge expected influence.

The node with the highest bridge EI in the network is ACSIConfidence and Achievement Motivation (bridge EI = 0.423), followed by General Recovery (bridge EI = 0.411), Recovery-stress balance (bridge EI = 0.277), and Anger (bridge EI = 0.242). This suggests that Confidence and Achievement Motivation play a significant bridging role in the network (Figure 4). Detailed Expected Influence and Bridge Expected Influence values are provided in Table 2.

**Table 2:** Centrality metrics for each node of the network.

node	Expected Influence	Bridge Expected Influence
Grade	−1.773	0.109
Coping with Adversity	0.151	0.019
Coachability	−1.489	0.105
Concentration	0.560	0.170
Confidence and Achievement Motivation	1.207	0.423
Goal Setting/Mental Preparation	0.996	0.181
Peaking Under Pressure	−0.442	−0.005
Freedom From Worry	−1.945	0.111
Emotion	0.308	0.021
Confidence	1.098	0.026
Anxiety	−0.366	0.021
Assertiveness	0.706	0.043
Cognitive	0.842	0.055
Bodily-somatic	0.095	0.121
Motor-behavioural	0.912	0.035
Operational	0.605	0.046
Communicative	0.521	−0.018
Social support	0.735	0.026
Anxiety	−1.231	−0.188
Dejection	0.967	0.209
Excitement	0.051	0.035
Anger	0.462	0.242
Happiness	0.257	0.204
General stress	−0.838	0.154
Sport Stress	−1.541	0.071
General Recovery	0.826	0.411
Sport Recovery	0.244	0.212
Recovery-stress balance	−1.915	0.277

Edges with strong Expected Influence in the network included:

Excitement vs. Happiness (weight = 0.747) and Dejection vs. Anger (weight = 0.535) in Sports Emotions. Assertiveness vs. Cognitive (weight = 0.534) in Sports Psychobiosocial Experiences. GS vs. Sport Stress (weight = 0.467) in Recovery-Stress. Anxiety vs. Dejection (weight = 0.418) in Sports Emotions. Edges with the strongest correlations with Competition Results included: Dejection (weight = 0.146), Sport Recovery (weight = −0.097), Confidence (weight = −0.019), and Goal Setting/Mental Preparation (weight = 0.048).

#### 3.3.3 Network accuracy and stability

**Figure 5:**
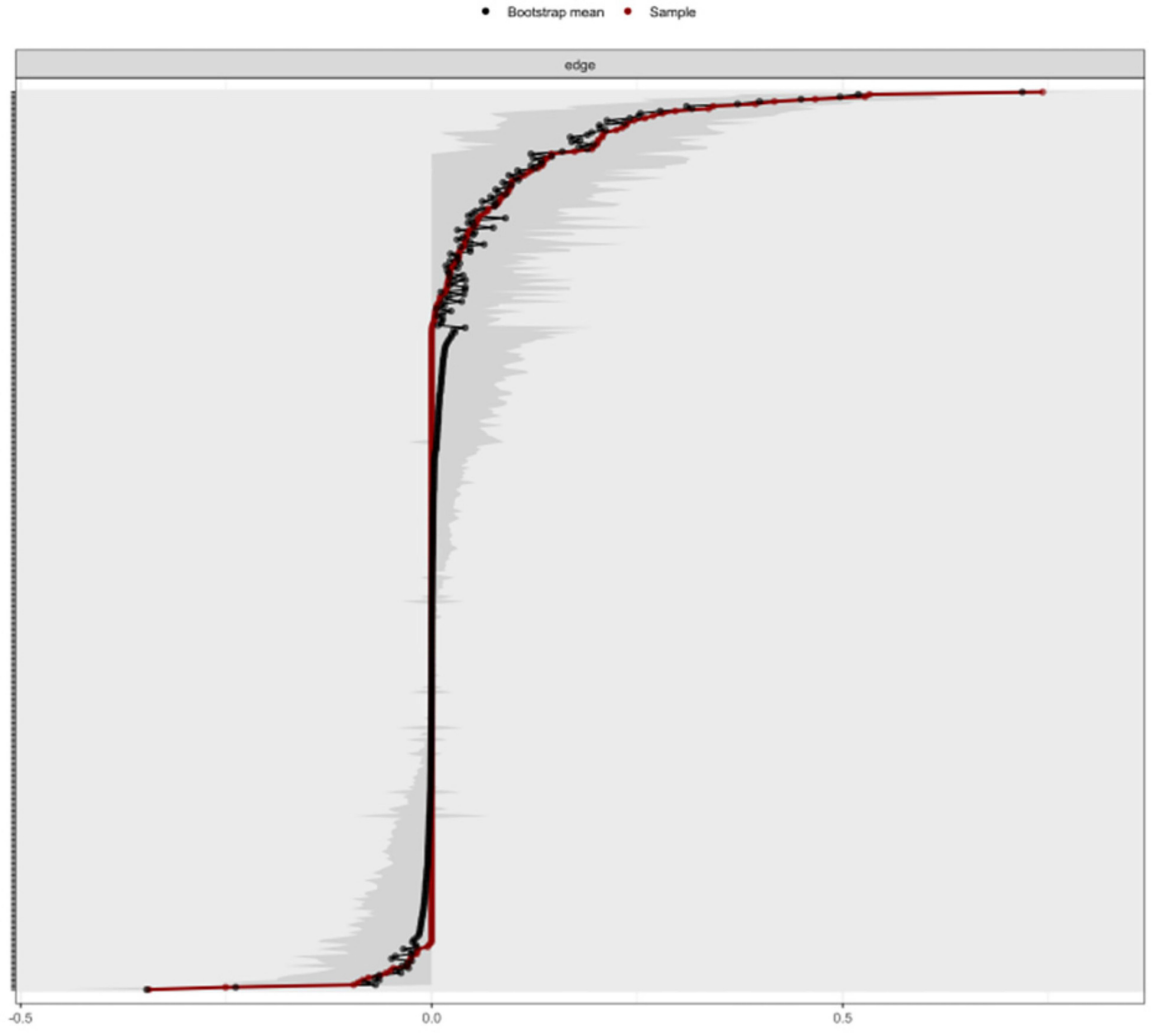
Bootstrap CIs for edge weights of dimensional networks. The red line represents the edge weight values and grey areas represent 95% CIs.

**Figure 6:**
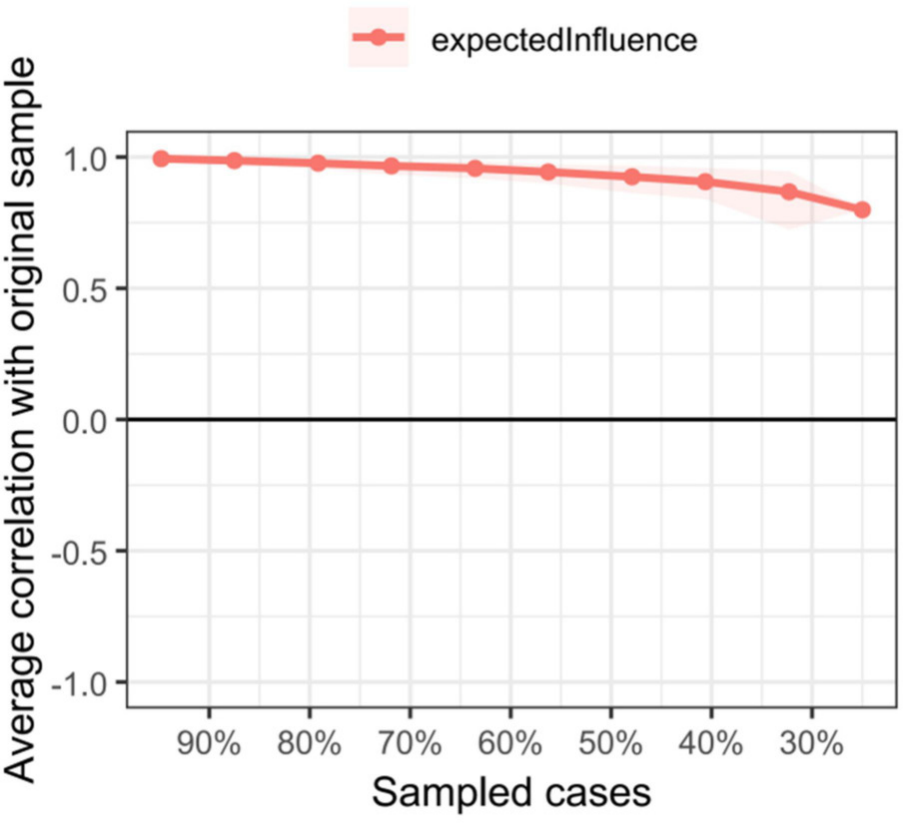
Bootstrap results for subsets of dimensional networks. The red line represents the marginal weights of the sample of this study; the black line represents the average marginal weights assessed by the self-help method; the grey area indicates the confidence interval derived by the self-help method.

The edge weight bootstrap results of the dimensional network (Figure 5) and the stability estimation of the centrality index (Figure 6) indicate that the dimensional network exhibits high accuracy. The network's Expected Influence (CS coefficients = 0.677 > 0.25) and Bridge Expected Influence (CS coefficients = 0.594 > 0.25) are stable and robust, demonstrating reliable coefficients. Accuracy analyses revealed that the edges of the network were accurately estimated, with each edge showing narrow CIs (Figure 5).

## 4 Results

In the network of dimensions, including Recovery-Stress, Sport Coping Skills, Sport Psychobiosocial Experiences, Sports Emotions, and Competition Performance in the conference system, Confidence and Achievement Motivation exhibited the highest Expected Influence Index (EI = 1.207) and served as the most central nodes among the dimensions. Confidence and Achievement Motivation play a crucial bridging role in the network, with the highest bridge EI (bridge EI = 0.423), and are key nodes connecting the network. Dejection, Sport Recovery, Confidence, and Goal Setting/Mental Preparation all showed significant correlations with Competition Performance, with Dejection being the most closely related.

## 5 Discussion

Confidence and Achievement Motivation is the nodes with the highest Expected Influence index and occupy a central bridging role in the network. Dejection, Sport Recovery, and Goal Setting/Mental Preparation were all associated with Competition Performance, with Dejection showing the strongest correlation.

### 5.1 Effect of confidence and achievement motivation on competition performance

In terms of the centrality indicator EI, ACSIConfidence and Achievement Motivation demonstrated the strongest centrality (EI = 1.207), followed by PESDConfidence (EI = 1.098), ACSIGoal Setting/Mental Preparation (EI = 0.999), and SEQDejection (EI = 0.967). These nodes hold central positions in the network and exert considerable influence on the overall system. The node with the strongest association between Confidence and Achievement Motivation in Sport Coping Skills and the other communities was General Recovery (weight = 0.24), which aligns with previous research findings. Positive coping strategies help athletes recover and should be emphasized during training and competition (52). The Confidence dimension in Psychobiosocial Experiences primarily showed a stronger connection within its own dimensions. Jones et al. (53) found that self-confidence is crucial for athletes. In the context of sports, it is essential for athletes to set goals scientifically and reasonably, while maintaining psychological adjustment in all aspects of their participation. Clearly defined goals provide athletes with a clear direction, increasing focus and motivation during training and competition, thus enhancing self-confidence (54). Adequate psychological preparation helps athletes maintain composure under competitive pressures and challenges, enabling them to perform optimally. Once athletes have carefully planned their goals and made sufficient psychological preparation, they often exhibit stronger self-confidence, surpass their limits, and strive for victory in competition. However, unrealistic goals may lead to disappointment and dejection (55).

In the network analysis of this study, ACSIConfidence and Achievement Motivation were found to be the nodes with the highest bridge EI (bridge EI = 0.423), indicating their key bridging role in the network. Specifically, Social support indirectly influenced General Recovery and Sport Recovery by linking to Confidence and Achievement Motivation in athletes’ coping skills. This result is partially consistent with previous research. Cosh and Tully (56) noted that access to Social support facilitated athletes’ recovery, but was also influenced by their coping skills. The present study aligns with this finding, further emphasising the bridging role of Confidence and Achievement Motivation. However, previous studies may not have explicitly identified the specific connecting mechanism of Confidence and Achievement Motivation between Social support and recovery, whereas this study reveals this critical pathway through network analysis. Self-efficacy theory (57) can explain this result. Confidence, as a key manifestation of self-efficacy, enables athletes to believe in their ability to handle various challenges, thereby enabling them to employ their coping skills more effectively in stressful situations. When faced with stress, athletes with higher Confidence and Achievement Motivation is more likely to actively seek and utilise social support, which, in turn, facilitates recovery. On the other hand, Achievement Motivation drives athletes to strive for excellence in performance, and to achieve their goals, they work to maintain good physical and mental health while actively utilising Social support to aid recovery. In conclusion, this study underscores the importance of focusing on and enhancing Confidence and Achievement Motivation in the development and training of athletes.

### 5.2 Competition performance

Competition performance was linked to dimensions in each variable (Recovery-Stress, Sport Coping Skills, Sport Psychobiosocial Experiences, Sports Emotions). The strongest correlation between Dejection and other communities in the node was Competition performance (weight = 0.146). A key consideration is the potential for suppressor effects or indirect pathways in the network. Network analysis reveals that Dejection is strongly positively correlated with Anger (weight = 0.535), a high-arousal negative emotion (32). demonstrated that anger can enhance muscle peak force and competitive drive when appraised as challenging (e.g., “I will use this frustration to outperform opponents”). In our network, Anger also shows a moderate positive correlation with Competition Performance (weight = 0.032). It is plausible that Dejection is a “collateral variable” linked to Anger, and the positive edge between Dejection and Competition performance is a byproduct of Anger's functional role—a classic suppressor effect where the direct negative impact of Dejection is masked by its correlation with a performance-enhancing variable (58).

Another potential confounder is task involvement and investment. Secondary school female volleyball players in the National Finals may experience dejection as a response to high personal stakes. This dejection could co-occur with increased effort investment, which is the true driver of Competition performance. Network analysis shows Dejection is positively correlated with Social Support (*r* = 0.287) and Confidence and Achievement Motivation (*r* = −0.266). Suggesting athletes experiencing dejection may seek more social support or double down on achievement goals to cope, indirectly boosting Competition performance.

The sample's unique characteristics, adolescent female volleyball players in a high-stakes team competition—may further explain this finding. Adolescents (average age = 17.26) are in a critical period of emotional regulation development, where negative emotions can sometimes trigger “compensatory effort” (52). Unlike individual sports, volleyball's team structure may buffer the direct negative effects of dejection: athletes experiencing dejection may rely on teammates’ support (reflected in the correlation with Social Support) or channel their sadness into collaborative effort. This aligns with Fogaca’s (16) finding that social support moderates the relationship between negative emotions and performance in team sports. Additionally, we emphasize the distinction between transient dejection (measured here as pre-competition emotion) and chronic dejection or depression. The SEQ assesses state-level dejection rather than trait-level hopelessness. Transient dejection, when paired with adaptive coping (e.g., Goal Setting/Mental Preparation, weight = 0.048 with performance), may serve as a “warning signal” that motivates athletes to address gaps in preparation—whereas chronic dejection would likely impair performance (59).

We acknowledge that our finding contradicts most prior research (34, 60), which links dejection to reduced focus, lower motivation, and poorer performance. This discrepancy may stem from: (1) the network analysis framework, which isolates partial correlations; (2) the specific competitive context (national-level team competition vs. individual or lower-stakes events); and (3) gender differences in emotional regulation (34), as female athletes may use negative emotions differently than male athletes. Future research should test whether this positive correlation is replicable in other samples (e.g., male athletes, individual sports) or if it is a context-specific phenomenon.

Sport Recovery and Competition performance had a marginal weight of (weight = −0.097), consistent with Skorski et al. (61) study, which found that athletes with good competition performance require more time for the recovery of sport-related bodily functions. Providing athletes with proper recovery conditions is essential to prevent poor performance during competition. The marginal weights for self-confidence and Competition performance were (weight = −0.019), contradicting Shuang and Xing (62) finding that male volleyball players with higher self-confidence have better competition performance. This may be related to gender, and different interventions targeting specific groups should be developed to foster athletes’ self-confidence, while also ensuring that overconfidence does not negatively affect performance. The side weight of the Goal Setting/Mental Preparation dimension of motor coping skills and Competition performance was (weight = 0.048), consistent with previous studies. Goal Setting has been shown to promote Competition performance (63), enabling athletes to perform better by setting desired goals for the competition. Additionally, Casanova and Meyers (64) found that interventions on motor coping skills can improve performance on the field, aligning with the present study's results. The findings suggest that recovery stress, motor coping skills, motor psychobiosocial experiences, and sports emotions each have varying degrees of influence on Competition performance, and that interventions targeting these psychological abilities can help adolescent female athletes perform better in competition.

### 5.3 Research limitations and future prospects

This study has several limitations. First, the sample size is relatively small (96 participants). Although it is somewhat reasonable for a preliminary exploratory study, for estimating a network structure containing 28 nodes, this sample size is relatively low and may affect the stability and generalisability of the network edge weights and centrality metrics, especially those with weaker associations. Future research could increase the sample size to further verify the reliability of the network structure and more comprehensively explore the impact of psychological abilities on the competitive performance of high school female volleyball players. Second, most of the data in this study were collected through self-report, which may have been subject to subjective bias. Future studies could collect objective indicators to enhance the credibility of the findings. Third, the study may have been influenced by external factors, such as social, cultural, and economic conditions, which could potentially affect the results but were not adequately considered in this study. Future research could broaden the scope by incorporating additional factors that impact the competition performance of high school female volleyball players. Fourth, the sample consisted of participants from one cohort of girls’ athletes in the National High School Volleyball Regional League Finals, which limits the external validity of the findings. Future research could explore factors influencing competition performance across athletes from various sports. In addition, this study employs a cross-sectional design, which can only reveal conditional associations between variables and cannot infer causality. Future research could further verify the causal effects between variables through longitudinal tracking or intervention studies.

## 6 Conclusions and recommendations

### 6.1 Conclusion

This study focuses on high school female volleyball players as a specific population, addressing the gap in existing sport psychology research that predominantly concentrates on adult or elite male athletes. By adopting network analysis as an innovative methodology (which provides a nuanced, systems-oriented perspective on athletes’ psychological profiles) and integrating multiple well-validated scales for comprehensive assessment of psychological and psychobiosocial variables, the study ensures the scientific rigor and reliability of its findings.

Confidence and Achievement Motivation emerged as the most central nodes in the network, representing key features of the association. In addition, Dejection, Sport Recovery, and Goal Setting/Mental Preparation were all linked to competition performance, with Dejection showing the strongest association. These findings indirectly suggest the potential importance of coping strategies, psychobiosocial experiences, sports emotions, and recovery-stress balance training in enhancing the competition performance of high school female volleyball players. The effects of Dejection may be the key to improving the competitive performance of these athletes.

### 6.2 Recommendations

Coping strategies, Psychobiosocial experiences, Sports emotions, and recovery-stress balance training can significantly improve the competition performance of high school female volleyball players. Therefore, this study recommends incorporating these strategies into volleyball training programs to diversify training methods and provide technological support for enhancing the competition performance of high school female volleyball players.Coaches play a central role in shaping athletes’ confidence, achievement motivation, and goal-setting behaviors. Coaches are encouraged to integrate psychological skills training into daily technical and tactical sessions, emphasize realistic and process-oriented goal setting, and create a supportive motivational climate that facilitates emotional regulation and adaptive coping.

## Data Availability

The original contributions presented in the study are included in the article/Supplementary Material, further inquiries can be directed to the corresponding author/s.

## Appendix A

Rankings of Girls’ Matches in the Finals of the 1st National Secondary School Volleyball Regional League

The 1st China secondary school volleyball regional finals placements of the female category.

**Table:**

Rank	Team	Score
1	SHANGHAI Athletics School	78
2	Ziyang Middle School of Sichuan Province	72
3	Tieli NO.1 Middle School	66
4	Chongqing No.8 Secondary School	60
5	Xuzhou Tongshan Tangzhang High School	54
6	Zhengzhou No. 9 High School	48
7	Wenzhou No. 2 Senior High School	42
8	No.1 Senior High School in Baotou	36
9	Beijing 101 Middle School	30
10	Hebei Sports Bureau Sports Technical School	24
11	Dalian Qiancheng Senior High School	18
12	Qitaihe TinJiBing senior high school	12
13	WEIFANG NO.7 Middle School	6
14	Taixing Experimental Middle School	0
